# Management of Women With an Unexpected Low Ovarian Response to Gonadotropin

**DOI:** 10.3389/fendo.2019.00387

**Published:** 2019-06-27

**Authors:** Alessandro Conforti, Sandro C. Esteves, Danilo Cimadomo, Alberto Vaiarelli, Francesca Di Rella, Filippo Maria Ubaldi, Fulvio Zullo, Giuseppe De Placido, Carlo Alviggi

**Affiliations:** ^1^Department of Neuroscience, Reproductive Science and Odontostomatology, University of Naples Federico II, Naples, Italy; ^2^ANDROFERT, Andrology and Human Reproduction Clinic, Campinas, Brazil; ^3^Department of Surgery, University of Campinas, Campinas, Brazil; ^4^Faculty of Health, Aarhus University, Aarhus, Denmark; ^5^GENERA, Centre for Reproductive Medicine, Clinica Valle Giulia, Rome, Italy; ^6^Medical Oncology, Department of Senology, National Cancer Institute, IRCCS Fondazione G. Pascale, Naples, Italy

**Keywords:** hypo-response, ovarian stimulation, Assisted Reproductive Technology, ovarian reserve, follicle-to-oocyte index, POSEIDON criteria, suboptimal response, ART calculator

## Abstract

POSEIDON groups 1 and 2 patients respond poorly (<4 oocytes retrieved) or sub-optimally (4–9 oocytes retrieved) to gonadotropin stimulation despite the presence of adequate ovarian parameters, which negatively affect their cumulative chances of delivering a baby using Assisted Reproductive Technology. A polygenic trait involving gonadotropins and/or their receptors seems to be the primary pathophysiology mechanism explaining this phenomenon. The clinical management is mainly focused on maximizing oocyte yield as to increase the likelihood of having at least one euploid embryo for transfer. Indices such as FORT (follicle output rate) and FOI (follicle-to-oocyte index) may be used to determine if the ovarian reserve was properly explored during a previous ovarian stimulation. Testing for the presence of common polymorphisms affecting gonadotropins and/or their receptors can also be considered to identify patients at risk of hypo-response. An individualized estimation of the minimum number of oocytes needed to obtain at least one euploid embryo can assist counseling and treatment planning. Among currently existing pharmacological interventions, use of recombinant FSH in preference over urinary gonadotropin preparations, FSH dosage increase, and use of rLH supplementation may be considered -alone or combined- for optimally managing POSEIDON's groups 1 and 2 patients. However, given the recent introduction of the POSEIDON criteria, there is still a lack of studies examining the role of interventions specifically to patients classified as groups 1 and 2, thus making it an area for open research.

## Introduction

The primary goal of Assisted Reproductive Technology (ART) is to provide effective and safe personalized solutions to help infertile couples obtain a live birth. This objective should be attained with the mindset of securing the shortest time to live birth while avoiding negative consequences for the mother and newborns. In this regard, the transfer of a single embryo at the blastocyst stage provides a higher implantation rate than the transfer of a cleavage stage embryo and limits the occurrence of multiple pregnancies (1–4). ART failure is indeed a leading cause of treatment dropout and is associated with an impairment of the psychological wellness of treated couples (5–7). Furthermore, the higher the number of ovarian stimulation (OS) cycles the higher the financial burden on couples, with potential long-term effects on general well-being (8). Thus, ART programs should strive to obtain a single live-birth using the least number of OS cycles as possible.

**Figure 1:**
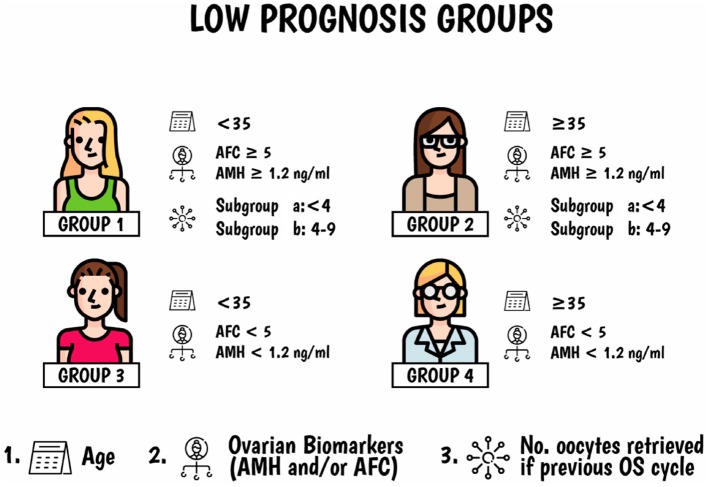
Poseidon criteria of low prognosis patients in ART. Four distinct groups of low prognosis patients can be established based on quantitative and qualitative parameters, namely: 1. The age of the patient and the expected embryo aneuploidy rate; 2. Ovarian biomarkers (antral follicle count [AFC] and/or anti-Müllerian hormone [AMH]), and 3. The ovarian response of the patient in terms of oocyte quantity provided a previous cycle of stimulation was carried out. Art drawing by Chloé Xilinas, EXCEMED, Rome, Italy. Adapted from Esteves et al. (10).

The novel Patient-Oriented Strategies Encompassing IndividualizeD Oocyte Number (POSEIDON) criteria (9–11) were introduced to help clinicians explore the possibility of using patient-oriented strategies to obtain the number of oocytes needed to achieve at least one euploid embryo for transfer in low prognosis women undergoing ART, as these patients represent the most vulnerable group concerning treatment failure and treatment dropout. A clear definition of the low prognosis patient population is, therefore, essential to avoid heterogeneity and allow the use of personalized management to achieve the intended goal. In brief, POSEIDON patients are subdivided into four subgroups based on a combination of factors including (i) age, (ii) results of functional and biological ovarian reserve markers, such as Antral Follicle Count (AFC) and Anti-Müllerian Hormone (AMH), and (iii) ovarian response concerning the number of oocytes retrieved in previous OS if this information was available (Figure 1). In practical terms, the POSEIDON criteria stratify the low prognosis patients in two main categories based on oocyte yield, namely, the “expected” low ovarian response (Group 3 and 4) and the “unexpected” low ovarian response (Groups 1 and 2).

POSEIDON's groups 1 and 2 encompass women who had poor (<4) or suboptimal (4–9) number of oocytes retrieved after a conventional OS despite the presence of an adequate ovarian reserve, defined by an AFC of ≥5 and/or an AMH ≥1.2 ng/mL. Indeed, retrieval of fewer than 10 oocytes is associated with decreased cumulative live birth rates (CLBR) (12). Among women with normal ovarian reserve, 10–15 oocytes seem to be the optimal target for increasing the likelihood of live birth rate in fresh embryo transfer (ET) cycles (13). However, retrieval of more than 15 oocytes might be advantageous concerning CLBR, i.e., when all fresh and frozen-thawed ETs are considered (12, 14). Thus, given a patient who fits POSEIDON's groups 1 or 2, the final goal would be to find ways to maximize oocyte yield aiming at obtaining more than 9 oocytes at the end of stimulation.

In this paper, we review the pathophysiology and discuss the available treatment strategies of low prognosis women according to POSEIDON's groups 1 and 2.

## Unexpected Suboptimal or Low Oocyte Number and its Association With Ovarian Hypo-response to Gonadotropin Stimulation

**Figure 2:**
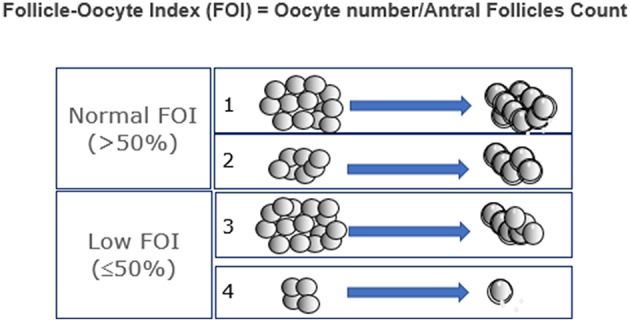
Ovarian resistance using the follicle-to-oocyte index (FOI). Reprint from Alviggi et al. (17).

**Figure 3:**
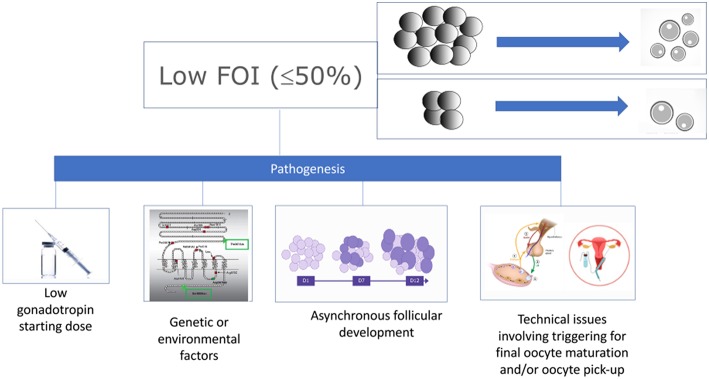
Pathogenesis of low follicle-to-oocyte index (FOI). Reprint from Alviggi et al. (17).

Patients who fit POSEIDON's groups 1 and 2 criteria should be critically assessed by looking at two indices, namely, the FORT (follicle output rate) and the FOI (follicle-to-oocyte index). The follicle output rate (FORT) measures the consistency between the pool of antral follicles at the beginning of OS and the number of pre-ovulatory follicles at the end of stimulation (15, 16). Along the same lines, the FOI assesses the consistency between the pool of antral follicles at the beginning of OS and the number of oocytes retrieved at oocyte pick-up (Figure 2). Thus, a discrepancy between the available antral follicle pool and the number of pre-ovulatory follicles at the end of stimulation (e.g., FORT <50%), or the number of retrieved oocytes (e.g., FOI <50%) is suggestive of hypo-response to gonadotropin stimulation, albeit other contributory causes might exist as depicted in Figure 3 (17). The advantages and shortcomings of using FOI and FORT to identify hypo-responders to gonadotropin stimulation have been discussed in detail elsewhere (17).

The pathophysiology mechanisms explaining the hypo-response to gonadotropin stimulation, also known as “ovarian resistance” to gonadotropin stimulation, are not fully understood. However, environmental contaminants, as well as specific genotypic traits have been hypothesized as possible contributory factors (17–21) (Figure 3). In particular, genetic polymorphisms affecting the gonadotropins and their receptors might impact OS outcomes (22, 23). Such polymorphisms include those affecting the FSH receptor genes, such as FSHR c.2039 A>G (rs6166) (24, 25), FSH β chain [FSHB−211 G>T (rs10835638)] (26), and FSH promoter region [FSHR−29 G>A (rs1394205)] (27, 28). Of particular interest is the FSHR polymorphism (rs6166), which has been implicated in ovarian resistance to exogenous gonadotropin (24). The single nucleotide polymorphism SNP (rs6166), known as Serine680 (Ser680) variant, causes the replacement of Asn with Ser at the 680 position and is located in the intracellular domain of the FSH receptor protein (29, 30).

Interestingly, it has been reported that Ser680 carriers with polycystic ovarian syndrome show resistance to clomiphene citrate (31). Another variant in a promoter region of FSHR, namely FSHR, 29 G>A), was associated with negative effects during OS. In a study by Achrekar et al. involving patients undergoing OS for ART, women homozygous for the rs1394205 variant genotype AA had a lower number of oocytes and lower pregnancy rates than those with the GG genotype (32). This observation was confirmed in a more extensive study by Desai et al. (27). These authors retrospectively evaluated 100 normogonadotropic women with regular menses who were candidates for IVF. The carriers of AA genotype showed a lower number of oocytes retrieved and a higher consumption of exogenous gonadotropin than GG carriers. As for the FSH β chain polymorphism (rs10835638), a study involving 169 healthy women, and 186 infertile women suggested that this polymorphism is associated with significantly higher FSH and LH levels in both healthy and female infertility patients (33). In this report, the T-allele carriers were found more frequently among idiopathic infertility cases, a fact that could be explained by the influence of this particular polymorphism on FSHR function (26, 34, 35).

A systematic review and meta-analysis published in 2018 summarized the data of 33 studies regarding the clinical relevance of FSHR polymorphism on OS. The authors showed that higher FSH consumption is expected in homozygotes for the A allele of the FSHR (rs1394205) polymorphism than in carriers of the G allele. Moreover, FSHR (rs6166) Serine carriers seem to be less responsive to OS treatment, with fewer oocytes retrieved at the end of OS than Asparagine carriers (22). In other words, both Serine carriers of FSHR (rs 6166) and A carriers of FSHR (rs1394205) are at increased risk of exhibiting ovarian resistance to OS, which in turn might lead to a suboptimal response concerning the number of retrieved oocytes at the end of OS.

Ovarian hypo-responsiveness to gonadotropin stimulation remains an undervalued issue in ART both in research and in daily clinical practice. Not surprisingly, the prevalence of this condition is still unclear. However, preliminary data show that ~10% of women defined as normal responders by demographic characteristics and ovarian reserve testing requires a higher than expected total dosage of gonadotropin to promote adequate follicular development (36). Notably, a recent study indicated that approximately 45% of patients aged 18–40 years who underwent conventional OS using FSH doses of 150–225 IU/day retrieved <10 oocytes at their first stimulation cycle (12, 37). These data may be overestimated as the authors did not specify the ovarian reserve before stimulation. By examining the data from a group of 427 consecutive infertile women who underwent conventional OS in one of the authors' (SCE) clinic, 47% of the treated patients fitted the POSEIDON criteria. Among them, 5 and 35% were within groups 1 and 2 categories, respectively (38). Although larger studies are required, these preliminary data suggest that a remarkable number of women with adequate pre-stimulation ovarian reserve parameters exhibits an unexpected anomalous response to gonadotropin stimulation.

## Clinical Management of POSEIDON's Groups 1 and 2 Patients

As highlighted above, there is a significant number of women classified as normal responders—based on ovarian markers—who show resistance to exogenous gonadotropin stimulation. Unlike those with diminished ovarian reserve in which the increment of gonadotropin dosage during ovarian stimulation appears to be of limited value (39), POSEIDON's groups 1 and 2 patients seem to benefit from a pharmacological intervention concerning the OS protocol. Given the possibility of the existence of a polygenic trait in POSEIDON's groups 1 and 2 patients, an option would be for clinicians to assess normal ovarian reserve patients concerning the most common polymorphisms in case of an unexpected poor (≤ 3 oocytes) or suboptimal response (4–9 oocytes) in previous IVF cycle. Ideally, if large randomized controlled trial confirmed the utility of pharmacogenomic approach, genotype screening could be potentially recommended even before ovarian stimulation avoiding inadequate OS attempts.

Patients with specific polygenetic traits would be at high risk of being classified as POSEIDON's groups 1 and 2 after conventional OS using empirical approaches. Thus, such patients could be identified *a priori* and treated accordingly using a pharmacogenomic rather than a trial and error approach. Indeed, pharmacogenomic algorithms have been used to evaluate how genetic differences among individuals might affect drug response, thus ultimately leading to the development of personalized drug therapies to compensate for these differences (30). The individual genomic variation could influence sensitivity to antimicrobials and response to cancer strategies (40).

Furthermore, genetic traits could influence fertility, including ovarian response to gonadotropin stimulation (41, 42), despite no obvious clinical signs or symptoms. These polymorphisms are widespread in population and women with infertile disorders (43). Considering that genotype analysis could be provided at lower cost compared with the past, it is plausible that a genotype mapping of women who showed an unexpected low response during OS could be of use to optimized management strategy in such women. Nevertheless, since the availability of polymorphism panel testing is still limited, another option would be to apply empirical pharmacological interventions to increase the oocyte yield—and eventually pregnancy outcomes—in such patients.

### Estimating the Number of Oocytes Needed to Obtain at Least One Euploid Blastocyst for Transfer

In 2016, the POSEIDON group introduced a new marker of success in ART, namely, the ability to retrieve the number of oocytes needed to obtain at least one euploid embryo for transfer (38). This marker represents a logical endpoint to guide clinicians develop an individualized treatment plan for ART patients, including those of POSEIDON's groups 1 and 2. Indeed, availability of a euploid embryo for transfer may change the fate of the low prognosis patient, as ~50–60% euploid blastocysts implant across all age categories (44). Thus, the higher the number of oocytes retrieved, the higher the probability of obtaining an embryo cohort that may include at least one euploid blastocyst (45, 46). However, retrieval of an optimal number of oocytes may not be feasible in patients of groups 1 and 2 due to reasons discussed in previous sections. The matter is further worsened by female age, which is the most critical predictor of embryo euploidy. In a recent study, using preimplantation genetic testing data of patients undergoing ART, we showed that blastocyst euploidy not only markedly decreases with female aging but also that the magnitude of decrease is progressive with every year of age (46). It would be therefore useful to estimate the individualized minimum number of oocytes needed to achieve at least one euploid blastocyst for transfer. A pretreatment predictive model—the ART Calculator- has been developed to assist clinicians to counsel and plan treatment regarding the number of oocytes required for at least one euploid blastocyst in IVF/ICSI procedures. Briefly, the model was constructed based on the results of the LASSO (Least Absolute Shrinkage and Selection Operator) regression analysis, which was utilized for both variable selection and regularization to enhance the prediction accuracy and interpretability of the statistical model. It is out of the scope of this paper to discuss the technical aspects of the calculator, but detailed information is provided in other papers within this Frontiers Research Topic. The “ART Calculator” is available online at http://www.groupposeidon.com/ and is fully aligned with the POSEIDON marker of successful outcome.

### Individualized Controlled Ovarian Stimulation

**Figure 4:**
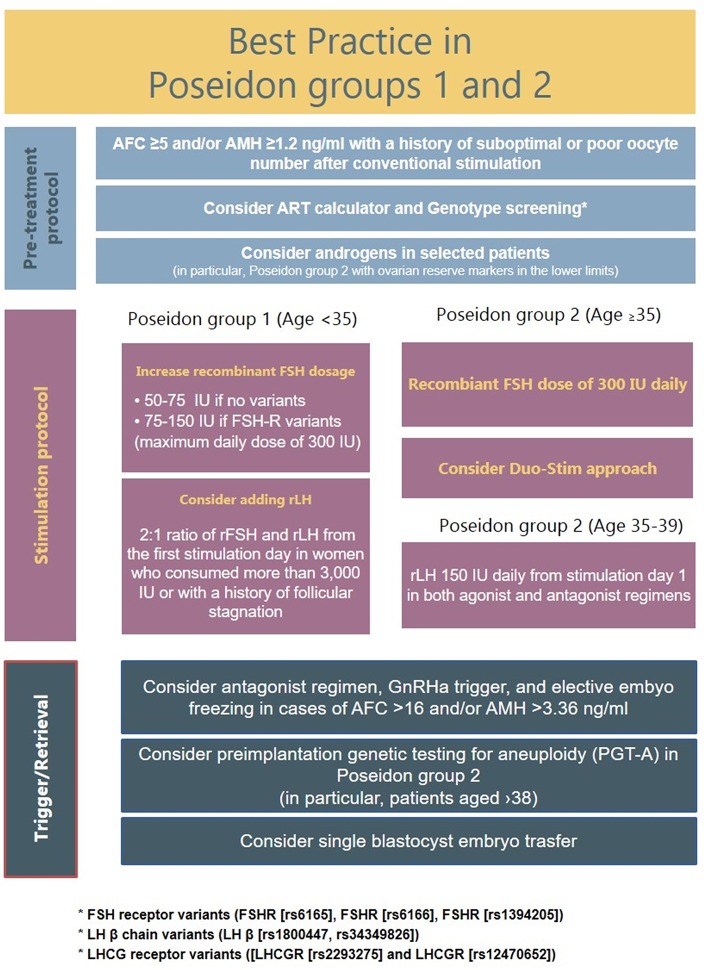
Suggested management of Poseidon groups 1 and 2 patients.

Based on limited data, five main strategies might be considered, which can be used alone or combined, namely (i) use of recombinant FSH in preference over urinary gonadotropin preparations, (ii) FSH dose increase, (iii) rec-LH supplementation, (iv) Dehydroepiandrosterone supplementation before OS, and (v) the combination of follicular and luteal phase stimulation in the same ovarian cycle. A flow chart listing the suggested management of Posedoin group 1 and 2 is illustrated in Figure 4.

#### Use of Recombinant FSH

The main problem behind suboptimal response or poor response is that the number of retrieved oocytes might not be consistent with the ovarian reserve. With the aim to retrieve more oocytes at the beginning of stimulation, a more “potent” gonadotropin formulation should be considered. Several randomized controlled trials and meta-analyses demonstrated that the use of recombinant formulations is associated with significantly higher number of retrieved oocytes than with urinary formulations irrespectively of the pituitary suppression strategy (47–49). These findings seem to relate to the higher bio-potency of recombinant formulations (50). In conclusion, the use of more potent (rFSH) recombinant formulation might be suggested in Poseidon Group 1 and 2.

#### FSH Dose Increase

Serine carriers of FSHR polymorphism undergoing OS for ART seem to benefit from increased recombinant FSH doses. In this regard, the first attempt to develop a pharmacogenomic approach to OS was conducted by Behre et al. (51). In their study, Ser680/Ser680 carriers were randomly allocated to two subgroups to receive a daily rec-FSH dose of 150 IU or 225 IU. The dose of 225 IU/day was able to restore the estradiol levels at the end of OS in Ser680/Ser680 carriers, which was similar to that of women with the wild-type genotype (51). Along the same lines, Genro et al. showed that when a high FSH dose (300 IU per day) was given, the FORT index was not significantly different in patients undergoing OS for ART, regardless of FSHR rs6166 genotype distribution (52). As for the FSHR rs1394205 polymorphism, we are unaware of any trials examining the effect of increased FSH doses. Furthermore, increasing FSH dosage appears to be a valid strategy in women with a history of suboptimal response *per se*. Specifically, a 2018 retrospective analysis evaluated the effect of FSH dose adjustment in women with a history of suboptimal response (4–9 oocytes retrieved) after conventional OS (37). In this study, 160 women <40 years with normal ovarian reserve undergoing their second ovarian stimulation cycle in a fixed gonadotropin-releasing hormone (GnRH) antagonist protocol with daily recombinant FSH (rFSH) were recruited. A dose increment of rFSH in the subsequent cycle carried out 4 months later on average lead to higher number of oocytes retrieved (9 vs. 6, *p* < 0.001) and good quality embryos (4 vs. 3, *p* < 0.001) than that of previous cycle. A regression analysis showed that an increase of 50 IU of the initial rFSH dose would lead to 1 more oocyte. Although there is evidence that resistance in term of the number of oocytes retrieved and follicle output rate could be associated with specific genotype anomalies, the suboptimal responders were not tested for genetic polymorphisms in this study.

#### Recombinant Luteinizing Hormone Supplementation

Several trials have examined the clinical utility of adding recombinant Luteinizing Hormone (rLH) in women with ovarian resistance to gonadotropin (53–58). In the larger ones, the ovarian resistance was identified in the form of an “initial slow response” in follicle growth (54, 55). In others, involving a lower number of cases, the hypo-response was retrospectively diagnosed in women who required higher-than-expected doses of gonadotropins considering their age, body mass index, and ovarian reserve (53, 56). Data from the more robust studies indicate that in slow responders, LH supplementation starting from stimulation days 7–10 might be more efficient than increasing the dosage of rFSH to rescue the ongoing cycle.

In detail, Ferraretti et al. study was a single-center randomized trial involving a total of 126 women aged 37 or younger undergoing pituitary suppression with the agonist protocol. The number of oocytes retrieved was significantly higher in hypo-responders treated with the rFSH plus rLH step-up regimen than in those who received a higher dose of rFSH (11.1 vs. 8.2, *p* < 0.05). Along the same lines, higher pregnancy rates per embryo transfer (54 vs. 24.4%, *p* < 0.05), live birth rates (40.7 vs. 22%, *p* < 0.005) and implantation rate (36.8 vs. 14.1%, *p* < 0.05) were observed in women supplemented with rLH than in those who received only an increment of rFSH. Another study was a multicenter RCT involving 229 IVF/ICSI cycles (55). The population, definition of hypo-response, and OS regimen were similar to Ferraretti et al. study. In this trial, the number of oocytes retrieved was significantly higher in patients who received rLH supplementation (9.0 ± 4.3) than in those treated with increased rFSH dosage (6.1 ± 2.6, *P* < 0.01). The use of rLH supplementation was able to restore both rescue implantation (14.2 vs.18.1%, *p* > 0.05) and ongoing pregnancy rates (32.5 vs. 40.2%, *p* > 0.05), which turned out to be resulted similar to that observed in normal responders. Regarding dosage, the use of 150 IU of rLH is apparently better than the use of 75 IU in the long GnRH agonist protocol (59). In a randomized trial, 46 hypo-responders identified by similar criteria as in Ferraretti et al. and De Placido et al. studies (54, 55) were randomized to receive a supplementation with 150IU or 75IU of rLH, respectively. Hypo-responders supplemented with 150 IU/day of rLH had higher number of oocytes retrieved (9.65 ± 2.16 vs. 6.39 ± 1.53, *p* < 0.05) and showed higher percentage of mature oocytes (79 vs. 65.7%, *p* < 0.05) than in those supplemented with 75 IU/day of rLH (59).

More recently, Yilmaz et al. (60) performed a single-center prospective study that corroborated the results of the randomized controlled trials mentioned above. In their study, hypo-responders were identified as in De Placido et al. study. A total of 137 patients were enrolled, 85 of whom had a hypo-response to OS diagnosed on stimulation on day 7 (at least six follicles between 6 and 10 mm; no follicle over 10 mm, and Estradiol levels below 180 pg/mL), and 52 had a normal response (regular follicular growth and Estradiol level >180 pg/mL). In the hypo-response group, 50 women received 75 IU daily of rLH, whereas the rFSH dosage was increased by 75 UI in the remaining 35. Implantation rates were significantly higher in controls (34.7%) and in the rLH supplementation (36.1%) groups than in the increased-dose rFSH group (15%, *P* < 0.02). The pregnancy rates were also higher in the two former groups than in the latter group (64.7 and 57.8%, respectively vs. 32.4%, *P* < 0.05). The findings of the studies mentioned above should be interpreted with caution because the GnRH agonist long protocol was utilized in all studies. Currently, there are no data concerning the use of rLH supplementation to hypo-responders undergoing OS under a GnRH antagonist regimen.

The mechanism by which rLH exerts its beneficial effect in hypo-responders is not fully understood. Although it was advocated that the excessive suppression of endogenous LH after down-regulation with GnRH analogs may create the need for exogenous LH supplementation, neither Ferraretti et al. nor De Placido et al. found a significant association between serum LH levels during OS and the response to rLH supplementation (54, 55, 61). A more plausible hypothesis would be related to genetically determined characteristics of LH itself or its receptors. Indeed, Alviggi et al. (36, 41) demonstrated that carriers of LH β chain variant had ovarian resistance to exogenous gonadotropin and required a higher dosage of recombinant FSH during OS (36). This variant was initially discovered by Pettersson and Söderholm (62) as an immunologically anomalous form of LH caused by two-point mutations in the β subunit gene, both altering the amino acid sequence (Trp8Arg and Ile15Thr). The LH variant has elevated bioactivity *in vitro* but significantly shorter (5–9 min) half-life in circulation than the wild-type LH (12–22 min) (63). This variant is common worldwide, with carrier frequency varying from 0 to 52% in various ethnic groups. Its incidence in Italy ranges between 12 and 13%. Another polymorphism that might be implicated in impaired ovarian response relates to those altering the Luteinizing hormone/human chorionic gonadotropin receptors (LHCGR) (64, 65). Specifically, a prospective cohort study investigated the effect of multiple gonadotropin polymorphisms on ovarian response in 94 normogonadotropic Caucasian women who underwent OS with a starting dose of 150 IU of recombinant FSH daily. In this study, the presence of allele C on both FSHR-min29 and LHCGR-291 was associated with an increased ratio between the cumulative r-FSH consumption and the total number of oocytes as well as mature oocytes (RR: 5.47, CI 95%: 3.13–7.81, *p* < 0.001) (65).

Lastly, a 2018 systematic review and a further meta-analysis evaluating the role of rLH in ART concluded that adding rLH to the stimulation protocol could be beneficial in two subgroups of patients, namely, (i) women with adequate prestimulation ovarian reserve parameters and an unexpected hyporesponse to rFSH monotherapy, and (ii) those with 36–39 years of age (57, 66). As discussed in the previous sections, many patients classified as POSEIDON's groups 1 and 2 will fit in the former subgroup. It seems, therefore, sound to consider adding rLH to OS. For them, 75–150 IU rLH can be started at the mid-follicular phase in an attempt to rescue the ongoing cycle or at stimulation day 1 in a subsequent cycle.

#### Dehydroepiandrosterone

Dehydroepiandrosterone (DHEA) supplementation before OS has been proposed to counteract the age-related fertility decline (67–69). A last Cochrane meta-analysis including 12 RCTs concluded that pretreatment with DHEA could significantly improve live birth rate in poor responders and in advanced age women (69). Two RCTs trials was conducted in women fitting Poseidon groups 2, namely those characterized by advanced age and good ovarian reserve (70, 71). In detail, Tartagni et al. conducted a RCT including 109 women between 35-40 years old with good ovarian reserve (i.e., AMH levels above 2 ng/mL). Patients recruited were assigned to DHEA supplementation (*n* = 53, 75 mg/die) or placebo (*n* = 56) eight weeks before OS. Higher live birth rate (22/53 vs. 18/56, *p* < 0.05) and lower miscarriage rate (0/53 vs. 5/56, *p* < 0.05) was observed in women supplemented with DHEA than in the placebo group. Similar findings was observed by Moawad et al. (71) in another RCT in which population study was randomized to receive DHEA (75mg/die) supplementation for 12 weeks before OS. Indeed, higher ongoing pregnancy rate (11/58 vs. 7/47, *p* < 0.05), was observed in women supplemented with DHEA versus no supplemented group. The rational behind the use of androgens could be related to the fact that an impaired theca function and androgens production is observed in advanced age women (72). Notably, it was observed that rFSH administration alone is not able to sustain androgens production in advanced age women (73). Furthermore, these findings corroborate the hypothesis that LH supplementation, which is the main regulator of theca cells, could be of use in advanced age women.

#### Double Stimulation in the Same Ovarian Cycle

A novel controlled ovarian stimulation approach has been proposed in women of low prognosis as a mean to increase the number of retrieved oocytes and the number of blastocyst available to biopsy for preimplantation genetic test for aneuploidies (PGT-A) in a single ovarian cycle (74). This method, referred to as “DuoStim,” combines the conventional follicular phase stimulation (FPS) with luteal phase stimulation (LPS). In both the FPS and LPS, patients undergo co-treatment with a maximal dose of rFSH (300 IU/day) plus rLH (150 IU/day) using a GnRH antagonist regimen (75) (Figure 4). The final maturation of oocytes in FPS and LPS was triggered by a subcutaneous bolus of buserelin (dose 0.5 mL) to reduce the time of luteolysis and the second stimulation started five days after the first retrieval. The DuoStim protocol might be considered a putative strategy in patients classified as Poseidon's groups 2 and 4, who are characterized by advanced maternal age. In this regards, a case-control study included 188 poor prognosis patients undergoing DuoStim with PGT-A, fitting at least two of the following conditions: poor ovarian reserve (i.e., AMH <1.5 ng/mg, AFC ≤ 6), advanced maternal age (≥35 years) and history of few numbers of metaphase II (MII) oocytes (≤ 5) demonstrated that oocytes/embryos derived from LPS showed similar oocytes competence as FPS-derived ones. Moreover, the authors provided evidence that on average more MII oocytes can be retrieved after LPS than after FPS (75, 76). Therefore, in these patients maximizing the number of oocytes would result in a dramatically higher opportunity to obtain a competent embryo per menstrual cycle in comparison to conventional stimulation. DuoStim was then explored in a large multicentre experience involving 310 women indicated to PGT-A, which confirmed comparable fertilization, blastocyst, euploidy, and pregnancy rates after euploid single-embryo-transfer oocytes/embryos from the FPS and LPS. In turn, the rate of patients obtaining at least one euploid blastocyst significantly increased from 42.3% (*n* = 131/310) after FPS-only to 65.5% (*n* = 203/310) with the contribution of LPS (77). Nevertheless, these results should be interpreted with caution since the treated patients did not explicitly fulfill the Poseidon groups 1–2 criteria. Thus, since promising, the use of DuoStim in Poseidon Groups 2 patients should be investigated further.

## Conclusions

Infertile patients undergoing ART may respond poorly (<4 oocytes retrieved) or suboptimally (4–9 oocytes retrieved) to gonadotropin stimulation despite the presence of adequate ovarian parameters. According to the new POSEIDON's criteria of low prognosis patients undergoing ART, they are classified as group 1 if younger than 35 years-old or group 2 if ≥35 years-old. Both groups are likely to have lower cumulative live birth rates than normal or high responders, an effect that is modulated by age. The pathophysiology mechanisms explaining this phenomenon are not fully understood but seem to be mainly associated with a polygenic trait involving gonadotropins and their receptors. The primary goal of management in POSEIDON's groups 1 and 2 patients is to maximize oocyte yield as to increase the likelihood of having at least one euploid embryo for transfer. For this, indices such as FORT (follicle output rate) and FOI (follicle-to-oocyte index) should be used to both identify the subset of hypo-responders and to determine if the ovarian reserve was adequately explored during a previous stimulation.

Moreover, testing for the presence of common polymorphisms affecting gonadotropins and/or their receptors might be considered in women belonging to Poseidon groups 1 and 2. Added to this, an individualized estimation of the number of oocytes needed to achieve at least one blastocyst for transfer-for instance, using the ART calculator- can make treatment more focused and cost-effective. Given the overlapping between POSEIDON's groups 1 and 2 categories and hypo-response to OS, several pharmacological interventions may be considered as regards clinical management of such patients. According to the best available literature, there are at least five strategies to be considered, which are not mutually exclusive, namely (i) use of recombinant FSH in preference over urinary gonadotropin preparations, (ii) FSH dosage increase, (iii) use of rLH supplementation, (iv) Androgens supplementation before OS, (v) DuoStim. Nevertheless, no trial explicitly examining the role of interventions to POSEIDON's groups 1 and 2 patients has been carried out yet. The introduction of POSEIDON criteria and practical indices such as FOI, along with polymorphism testing, could help to understand better this specific subgroup of patients undergoing ART. Also, such an approach can be used to design robust clinical trials aiming at finding the optimal clinical management, thus making it an area open for further research.

## Author Contributions

AC, CA, and SE designed the manuscript and scrutinized the literature. All authors contributed to drafting and critical discussions. All authors contributed to revised and accepted the final manuscript.

### Conflict of Interest Statement

AC, CA, SE, FU, and GD are co-founders and members of the POSEIDON group. CA received honoraria for lectures from Merck. SE received honoraria for lectures from Merck, Besins, Lilly, and Gedeon-Richter. The handling editor is currently co-organizing a Research Topic with two of the authors SE and CA, and confirms the absence of any other collaboration. The remaining authors declare that the research was conducted in the absence of any commercial or financial relationships that could be construed as a potential conflict of interest.
